# The Acoustic Emission Method Implementation Proposition to Confirm the Presence and Assessment of Reinforcement Quality and Strength of Fiber–Cement Composites

**DOI:** 10.3390/ma13132966

**Published:** 2020-07-02

**Authors:** Anna Adamczak-Bugno, Aleksandra Krampikowska

**Affiliations:** Faculty of Civil Engineering and Architecture, Kielce University of Technology, Aleja Tysiąclecia Państwa Polskiego 7, 25-314 Kielce, Poland; akramp@tu.kielce.pl

**Keywords:** cement–fiber boards, acoustic emission method, k-means algorithm, wavelet analysis, fiber composites

## Abstract

This article proposes to use the acoustic emission (AE) method to evaluate the degree of change in the mechanical parameters of fiber–cement boards. The research was undertaken after a literature review, due to the lack of a methodology that would allow nondestructive assessment of the strength of cement–fiber elements. The tests covered the components cut out from a popular type of board available on the construction market. The samples were subjected to environmental (soaking in water, cyclic freezing–thawing) and exceptional (burning with fire and exposure to high temperature) factors, and then to three-point bending strength tests. The adopted conditions correspond to the actual working environment of the boards. When applying the external load, AE signals were generated, which were then grouped into classes, and initially assigned to specific processes occurring in the material. The frequencies occurring over time for the tested samples were also analysed, and microscopic observations were made to confirm the suppositions based on the first part of the tests. Comparing the results obtained from a group of samples subjected to environmental and exceptional actions, significant differences were noted between them, which included the types of recorded signal class, the frequency of events, and the construction of the microstructure. The degradation of the structure, associated with damage to the fibers or their complete destruction, results in the generation under load of AE signals that indicate the uncontrolled development of scratches, and a decrease in the frequency of these events. According to the authors, the methodology used allows the control of cement–fiber boards in use. The registration and analysis of active processes under the effect of payloads makes it possible to distinguish mechanisms occurring inside the structure of the elements, and to formulate a quick response to the situation when the signals indicate a decrease in the strength of the boards.

## 1. Introduction

Fiber–cement is a natural material that is widely used in construction. Its creator was Czech engineer Ludwig Hatschek. At the beginning of the last century, to produce such elements, asbestos was used. In the 1970s and 1980s, asbestos cement sheets were popularly called eternit [1,2,3,4,5].

Since then, the method of fiber–cement production has undergone many transformations. First of all, asbestos has been eliminated and replaced with completely harmless materials. The appearance of fiber–cement products has also changed significantly [6,7,8].

The relatively high strength parameters of fiber–cement boards are the result of their production process. The components are made of Portland cement (90%) and rayon staple (10%). Cement binds the material and determines its final strength. Cellulose provides the right amount of water in the cement setting process, fills the gaps and increases the density of the product. It also serves as the reinforcement. In the production process, mineral materials are also used as fillers to improve the flexibility and appearance of the boards [9,10,11,12,13].

The fiber–cement production technology consists of applying successive thin layers of a mixture of materials, which are pressed well together before the slow hardening process. The applied technological process allows fiber–cement to achieve high strength parameters [14,15,16].

As mentioned above, boards can be used both inside and outside buildings. Installation of the components in an outdoor environment involves exposure of the boards to weather conditions. Therefore, it is considered appropriate to perform tests on fiber–cement components taking into account the conditions to which they will be exposed during use. Among the typical interactions, the authors indicate atmospheric precipitation and the recurring process of freezing and defrosting the boards. Certain factors, specified in the standard guidelines, should be taken into account when testing the physical and mechanical parameters. Attempts to evaluate the condition of fiber–cement components must also take into account the likelihood of special factors, which include high temperatures and direct fire [17,18,19,20].

The paper presents the application of the acoustic emission (AE) method, based on unsupervised pattern recognition (k-means algorithm), to evaluate the change of the mechanical parameters of fiber–cement components. Three-point bending tests were performed on material samples in an air-dry state, which were then subjected to environmental (soaking in water, and recurring freezing and defrosting) and special (flaming with a gas burner and exposing to a high temperature—230 °C for 180 min) operating factors. According to the authors, the environmental factors listed above correspond to the typical operating conditions of external cladding boards, which must always be taken into account in the test procedures. The flame action reflected the fire conditions that impact directly on the partition covered with cement–fiber boards. The high temperature was also intended to reflect fire conditions (range of the high temperature zone) and heat accumulation in the material. The recorded AE signals (generated by active developing destructive processes) were divided into four classes. Each of them reflects certain processes taking place in the structure of components under an external load. The frequencies occurring over time in each of the tested samples were also analysed.

After the three-point bending strength test, fragments of fractures were extracted from the tested components for microstructure tests, performed with a scanning electron microscope. Parallel to the observations of the fracture surfaces, an analysis of the elemental composition of the material was performed using the EDS (Energy Dispersive X-Ray Spectroscopy) microprobe.

The authors undertook the indicated research topics because they believe that the use of the AE method may enable the assessment of the condition of the cement–fiber boards in operation. So far, most of the research on cement–fiber boards has been devoted to the influence of operational factors [21,22] and the action of high temperatures, examined by testing some of the physicochemical parameters of the boards, especially their bending strength (Modulus of Rupture MOR)). The nondestructive testing of fiber–cement boards was mainly limited to detecting imperfections arising at the production stage. Articles by Drelich et al. [23] and Schabowicz and Gorzelańczyk [24] present the possibility of using Lamb waves in a non-contact ultrasonic scanner to detect such defects. In the literature on the subject, there is little information on the use of other nondestructive testing methods for fiber–cement boards. The research described in the works of Chady et al. [25] and Chady and Schabowicz [26] showed that the terahertz (T-Ray) method is suitable for testing fiber–cement boards. Terahertz signals are very similar to ultrasonic signals, but their interpretation is more complicated. In [27], the microtomography method was used to identify delaminations and low-density areas in fiber–cement boards. Test results indicate that this method clearly reveals differences in the microstructures of the elements. Therefore, the microtomography method can be a useful tool for testing the structure of fiber–cement boards, in which defects may arise due to manufacturing errors. However, this method can only be used for small size boards. It should be noted that, so far, few cases of testing fiber–cement boards using acoustic emissions have been reported. Ranachowski and Schabowicz et al. [27] conducted pilot studies on fiber–cement boards manufactured by extrusion, including exposing boards to 230 °C for 2 h. In this research, an acoustic emission method was used to determine the impact of cellulose fibers on boards’ strength, and they attempted to distinguish between the AE events emitted by the fibers and the cement matrix. The results of these tests confirmed the usefulness of this method for testing fiber–cement boards. In [28], by Gorzelańczyk et al., the use of the acoustic emission method was proposed to examine the effect of high temperatures on fiber–cement boards. It should be noted that the effect of high temperatures on concrete, as well as the interrelationships associated with this process, have been extensively described using the acoustic emission method; an example is Ranachowski’s work [5]. AE was used to assess the state of concrete-like material and consider the effects of extreme conditions on it, such as fire or frost (papers [29,30,31,32]). It should also be mentioned that the acoustic emission method is often used to test thin materials, e.g., steel and polymer composites, and even fragile food products [15,33]. Nondestructive tests have also been used to show decay, and the correlation between static and energy performances [34].

Analysing the results of the conducted works [35,36,37,38,39], it can be stated that for cement matrix panels reinforced with fibers, the biggest threat is the damage or degradation of the reinforcing fibers, as well as any decrease in the degree of binding between the matrix and reinforcement, as these decrease the mechanical parameters of the composites. In turn, taking into account the fact that these panels are mounted on facades of public buildings classified as high, a decrease in the strength of cladding elements may be associated with a real threat to human health and life. On the other hand, the wide applicability of the acoustic emission method suggests that it will also give positive results when assessing the condition of fiber–cement boards.

To sum up, it has been found that there is no methodology in the existing literature for assessing the condition of fiber–cement boards. For this reason, the authors conducted research using the acoustic emission method, the results of which clearly show that the signals recorded for samples with high mechanical parameters differ significantly from those for elements with a degraded structure and reduced strength. The differences consist in the number of recorded signals, belonging to classes corresponding to the destructive processes occurring in the material, as well as the frequencies emitted.

## 2. Materials and Methods

The tests were performed on rectangular samples cut out from a 3100 × 1250-mm fiber–cement board. Information about the components intended for the three-point bending strength test is presented in Table 1.

**Figure 1:**
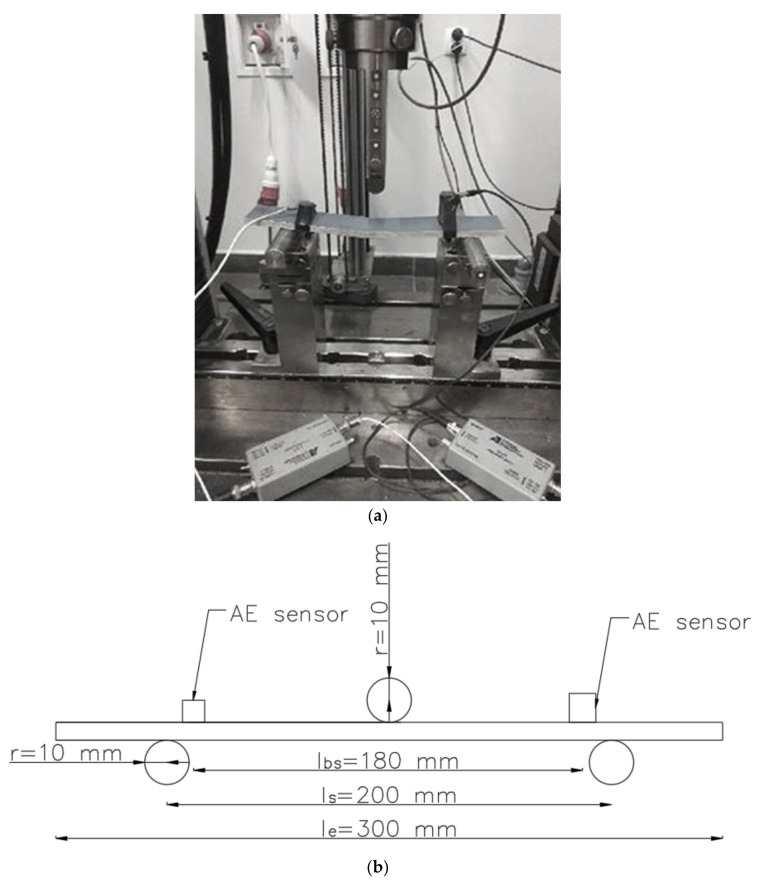
The fiber–cement element in three-point bending with installed AE sensors: (**a**) photograph of the specimen; (**b**) scheme of loading and AE sensors.

The tests were performed using a Zwick Roell universal testing machine. The bending speed for each sample was 0.1 mm/min (each test was carried out with a constant increase in deflection). The scheme of the test stand is shown in Figure 1. The axial distance between the support points *ls* was 200 cm, and the radius of the support points and the loading beam *r* was 10 mm. Based on the measurement data, the *MOR* bending strength of the elements was calculated in accordance with EN 12467 Fibre-cement flat sheets - Product specification and test methods.

During each test, acoustic emission signals were recorded. The eight-channel Vallen AE processor board was used for this purpose. The acquisition was carried out using two sensors with built-in preamplifiers—VS30-SIC (25–80 kHz range) and VS150-RIC (100–450 kHz range). During the tests, 13 typical AE parameters (AE signal duration, AE rise time, mean effective voltage, number of counts, number of counts to maximum signal amplitude, amplitude of AE signal, signal energy, average frequency, reverberation frequency, initiation frequency, absolute energy of the AE signal, signal strength and average signal level), and the values of strength increase and deformation of the samples, were recorded. The sensors were attached with a clamp near the supports on the inside.

Analysing the literature, it can be assumed that the course of the destruction of ordinary concrete (mortar, cement matrices) under short-term loading is three-stationary. These stages are stable microcracks initiation, stable microcracks development and propagation, and unstable microcracks propagation.

The stage of stable crack initiation is characterised by microcracks, that were initiated already at the stage of this material’s formation, appearing at isolated points of the concrete material, in the form of micro-gaps, pores and local concentrations of tensile stresses. The formation of these microcracks alleviates existing stress concentrations, leading to the restoration of the balance of internal forces. It is characteristic that at this stage of destruction, the existing microcracks do not develop, while the phenomenon of their increase occurs.

The increase in load causes the destruction of concrete to enter the second stage, in which two simultaneous processes occur: the phenomenon of crack propagation created in the first stage, and the further formation of stable microcracks. The cracks multiply and spread in a stable manner, in the sense that if the external load increase is stopped, the development of the cracks will also cease.

The third, final stage occurs when, as a result of a further increase in load, the crack system develops to such an extent that it becomes unstable. Under the influence of the released energy of deformation, the cracks spread automatically until the structure is completely destroyed. Destruction at this stage can occur even without further increase in external load.

The works of Kanji Ono, Othsu and Fowler [40,41,42,43] state that the sources of the AE signals in elements made of a concrete (cement) composite can be:

- The creation of microcracks;
- crack formation and propagation;
- cracks closing (friction at the concrete–concrete border);
- friction at the concrete–reinforcement border;
- corrosion, erosion;
- plasticisation and destroying of the reinforcement.

The recorded signals were divided into classes using the k-means algorithm, using Vallen software. In the course of the procedure, a standard sample was selected, in which, during the bending process, all the processes characteristic of the fiber–cement material were generated. The standard file was used to divide the signals recorded for the remaining samples.

**Figure 2:**
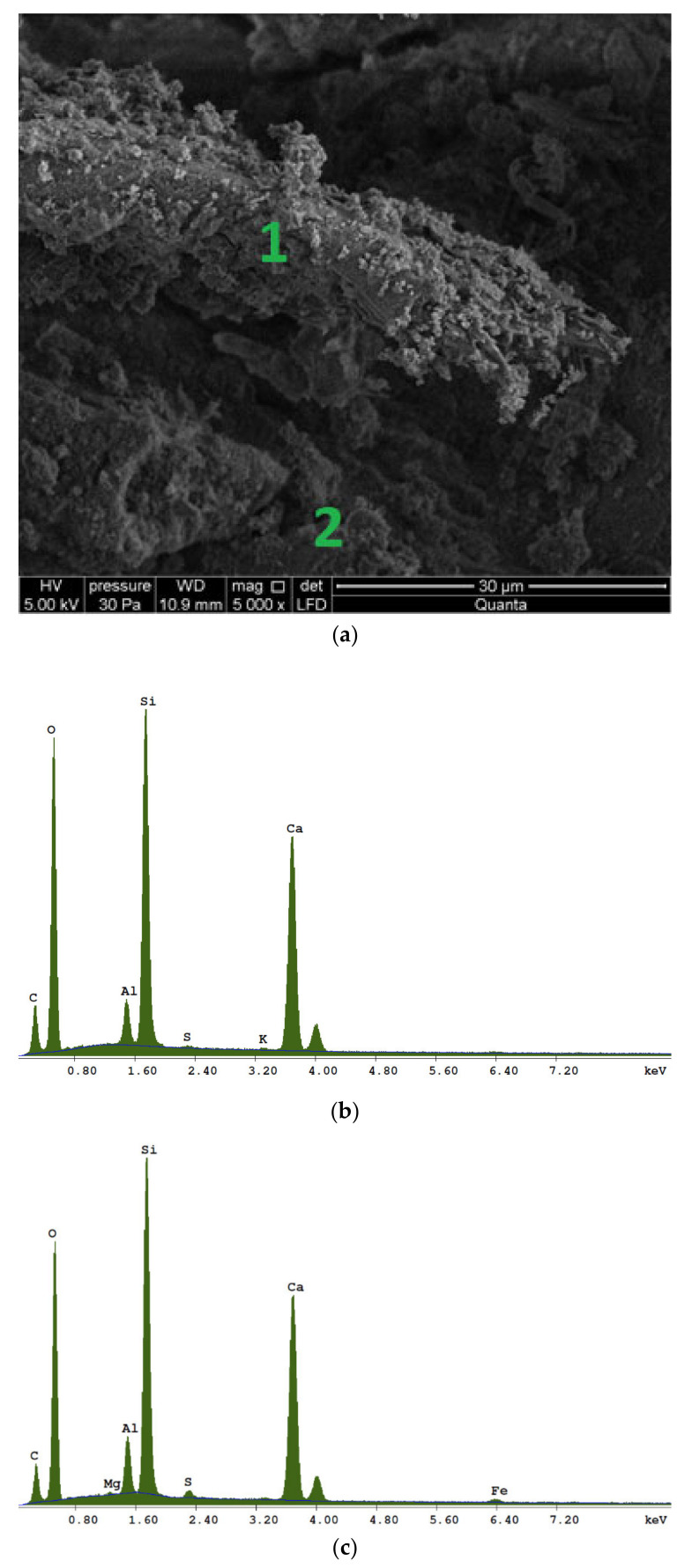
EDS analysis results for a mock sample: (**a**) distribution of measuring points; (**b**) analysis results from point 1—fiber; (**c**) Analysis results from point 2—matrix.

The test components came from the type of fiber–cement board that is available on the construction market, which has a broad application range (installed inside and outside buildings). As declared by the manufacturer, the samples contained Portland cement, mineral binders, natural organic reinforcing fibers and synthetic organic reinforcing fibers. Elemental composition was confirmed by performing analyses using an EDS micro probe during microscopic observations (Figure 2). The comparison of the data provided by the manufacturer with the EDS analysis performed is presented in Table 2. The analysis of the elemental composition of the matrix and fiber allowed us to assess the type of binder used. In the previous research on fibrous cement materials carried out by the authors, it was found that cement matrices are made using only Portland cement, or Portland cement with the addition of other mineral binders. Performing such an analysis in this case gave the information that Portland cement and a silicate mineral binder were used in the production of the matrix. Technical data from the tested boards are presented in Table 3.

Microscopic observations were performed after the strength tests, and were combined with the AE signal generation. A 10 × 10-mm piece was cut out from the fracture surface of each sample. The components placed in the microscope chamber had not been sprayed before. The observations were performed at 5 kV with an LFD (Low vacuum Secondary Electron) detector designed for operation in low vacuum.

### 2.1. K-Means Algorithm in the AE Method

To build the base of reference signals, used to assess the change of mechanical parameters in cement–fiber composites, the pattern recognition method was used, specifically, the version with arbitrary division into classes (unsupervised)—USPR. Arbitrary pattern analysis is mainly used when creating a database of reference signals if the number of classes is unknown. The k-means grouping method was used to divide the signals into classes corresponding to the destructive processes in cement composites.

K-means is a standard cluster analysis algorithm, in which the value of parameters determining the number of groups to be extracted from a data set is initially determined. Representatives are randomly selected, so it is important that they are as far apart as possible [44,45,46,47]. The selected components are the seedbed of the groups (prototypes). In the next step, each component of the set is assigned to the nearest group. Initial groups are designated at this stage. In the next step, a centre is calculated for each group, based on the arithmetic mean of the coordinates of the objects assigned to a group. Then, all the objects are considered and reallocated to the nearest (with respect to their distance from individual centroids) group. New group centres are designated until the migration of objects between clusters ceases. According to the same principle, the assignment correctness of particular objects to particular groups is checked. If in the next two runs of the algorithm there is no change in the division made (at which point it is said that the stabilisation is achieved), the processing is finished [48,49,50]. In this method, the number of groups is constant and consistent with the *k* parameter; only the group object assignment can be changed. In the k-means method, the search for the optimal division corresponds to the designation of the prototypes of groups that minimises the following criteria function [51,52]:

(1) $$J_{\left(v,B\right)}=\sum_{i=1}^{k}\sum_{k=1}^{N}b_{ik}d^{2}\left(v_{i},v_{k}\right),$$

In this function, *d(v,x)* is the distance of the element represented by the vector *x* from the group designated by the prototype (centroid, centre of the group) *v, N* is the number of the set *O, B* is the division matrix, and the other parameters have the same meaning as stated above. The principle of the method can be described as follows:

1. Preliminary division of the set into *k* clusters;
2. Calculation of an individual centroid for each cluster (centre of the group);
3. Assigning of each element of the set to the nearest group (in this case, the distance from the group is the same as the distance from the centroid);
4. Repetition of the previous two steps until the changes related to the object assignment to clusters ceases.

Creating a base of reference signals involves several stages. These are:

- generating signals in the laboratory while destroying specially designed samples in a certain way;
- comparison of signals received from samples with signals generated during the destruction of model beams subjected to various destruction processes;
- verification of reference signals on the basis of monitoring results of various types and lengths of composite panels loaded for destruction;
- final verification on the elements during their normal operation (this stage will be carried out in subsequent tests).

The characteristics of the signals contained in the database include the geometric, energy and frequency parameters of the signals. In addition, the database contains typical noise signals.

It should be emphasised that the main purpose of the research was to register the AE signals generated by destructive processes. Therefore, no additional tests, e.g., deformation of concrete under compression, or measurement of Young’s modulus, were carried out, and no statistical calculations were carried out regarding the spread of parameters. This approach was derived from the fact that the actual element lacks information about the state of the concrete at the test site, and the purpose of the research was to collect the most real AE signals generated during the destruction of the tested samples.

A total of 13 AE parameters was used to create the base of reference signals for the destructive processes that take place in cement–fiber boards:

1. AE signal duration (duration);
2. AE rise time (rise time);
3. Mean effective voltage (RMS);
4. Number of counts;
5. Number of counts to maximum signal amplitude (counts to peak);
6. Amplitude AE signal (amplitude);
7. Signal energy (energy);
8. Average frequency AE (average frequency);
9. Reverberation frequency;
10. Initiation frequency;
11. Absolute energy of the AE signal (absolute energy);
12. Signal strength;
13. Average signal level AE (ASL).

The second step was the adoption of the basic parameters necessary to create a base of reference signals, namely:

- expected number of destructive processes (based on literature and our own research);
- measures of distance between clusters—in this case, the Euclidean distance with time distribution;
- the number of iterations needed to find the optimal number of classes (minimum 1,000,000).

The standard file, with four classes obtained in this way, was then checked with real samples in laboratory tests of individually destructive processes.

The result of the research was the obtaining of a base of reference signals intended for the assessment of the technical condition of the boards. Using the base of reference signals, individual AE parameters (individual graphs) are only an illustrations of the processes taking place, not a source of analysis. That is why it is so important to use BIG DATA analysis to create “BLACK BOX”, which can be used by persons without any academic knowledge of AE to analyse the technical condition of the structure.

In the presented research, the issue of signal localisation was not addressed. This issue will be addressed in further studies on full-size elements. So far, the focus has been on identifying active destructive processes.

### 2.2. Scanning Electron Microscopy with Elemental Composition Analysis

Scanning electron microscopy (SEM) allows the recording of a surface image of samples at high magnification, by means of secondary or backscattered electron recording. Unlike optical microscopy, it allows for much higher magnifications, with incomparably higher resolution. The electron beam surface scanning of samples is a simple and quick way to obtain images that reflect differences in the elemental composition of the sample [53,54,55]. Accessories, such as the EDS microprobe analyser, allow for quick elementary composition determination at points and areas of different sizes. The element identification is based on the recording of the X-ray energy spectrum emitted by the sample atoms induced by the electron beam. The software automatically determines the elementary composition of the sample based on the characteristic radiation. The combination of electron microscopy with elementary analysis allows us to record high-resolution images at very high magnifications, determining the elementary composition of very small objects (even less than 1 mm), and creating change profiles for the composition of components and two-dimensional colour maps of element distribution on samples’ surfaces. The method is considered to be non-invasive as the destructive effect of the electron beam on samples is very rare, and, in addition, such effect occurs (if it occurs) on a microscopic scale. The scanning electron microscope does not require the spraying of conductive layers on the surface of the materials to be tested, thanks to a special measurement mode at low pressure (called low vacuum measurement) [56].

## 3. Results

### 3.1. Results of AE Signal Analysis

#### 3.1.1. Individual AE Signal Class Distribution Analysis

After dividing the recorded AE signals into four classes, using k-means algorithms, two AE parameters were analysed (as an illustration of ongoing processes)—signal strength and signal duration. The number of classes was input by the authors into the software used to analyse the AE signals. The value adopted corresponds to the nature of the material’s work, and allows us to link individual classes with specific processes. Another criterion explaining the imposed number of classes was the level of individual signals matched to the appropriate classes, which in this case was over 90%. The following individual AE signal classes were assigned to processes occurring in the structure of the tested material:

- class 1 (green)—formation of microcracks;
- class 2 (red colour)—crack development;
- class 3 (yellow)—cracking of reinforcing fibers, delamination of the structure, detaching the fibers from the matrix;
- class 4 (colour blue)—breakdown of reinforcing fibers, sample destruction.

The paper presents graphs of the descriptors over time, including their division into classes, for mock samples from each of the tested series. The results obtained for samples within the group were similar.

**Figure 3:**
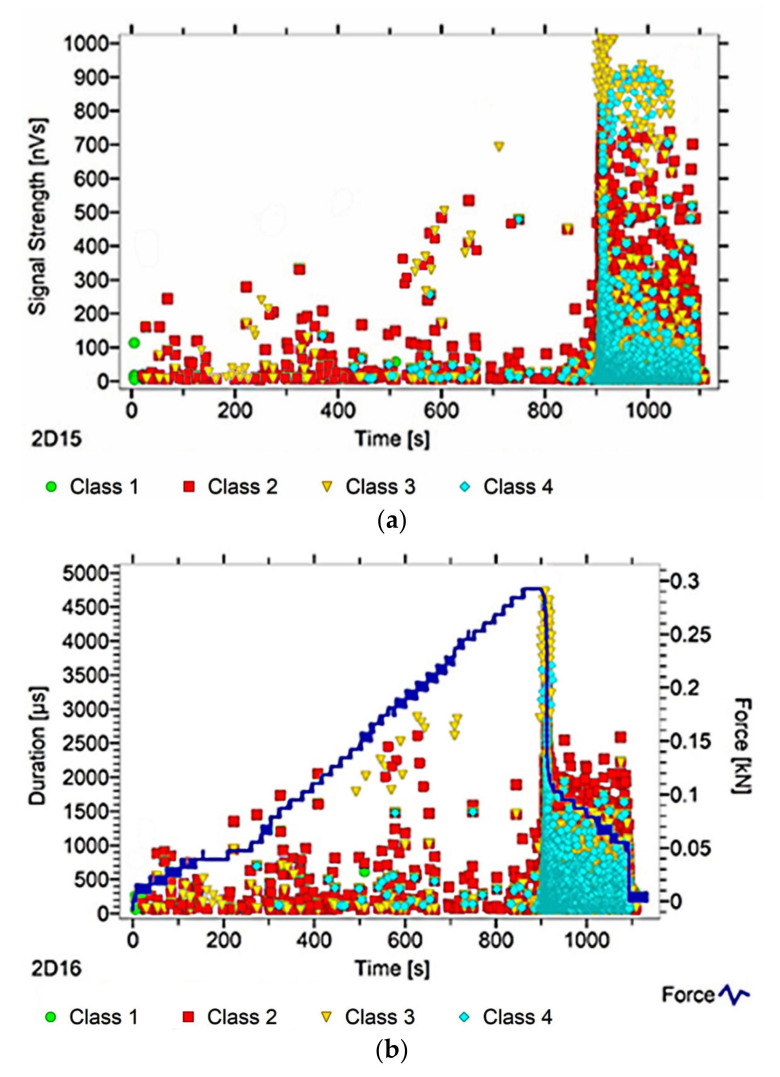
AE signal graphs for the mock BS sample: (**a**) signal strength distribution in time; (**b**) signal duration distribution in time, with plotted force increment curve.

**Figure 4:**
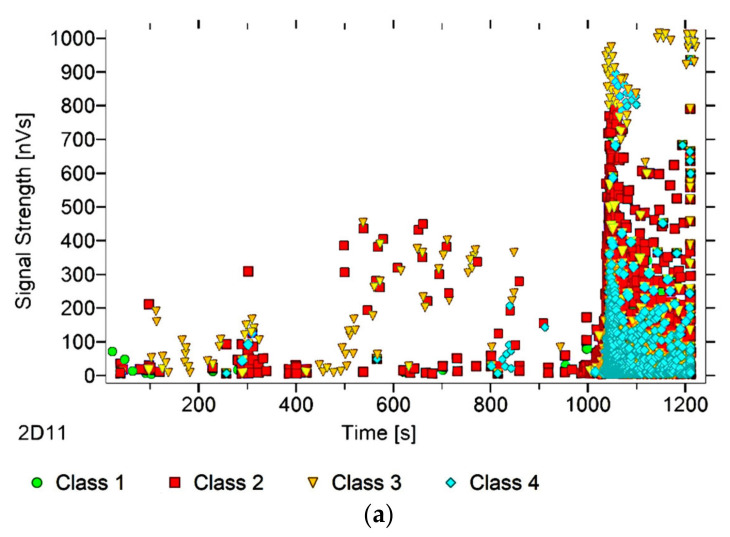

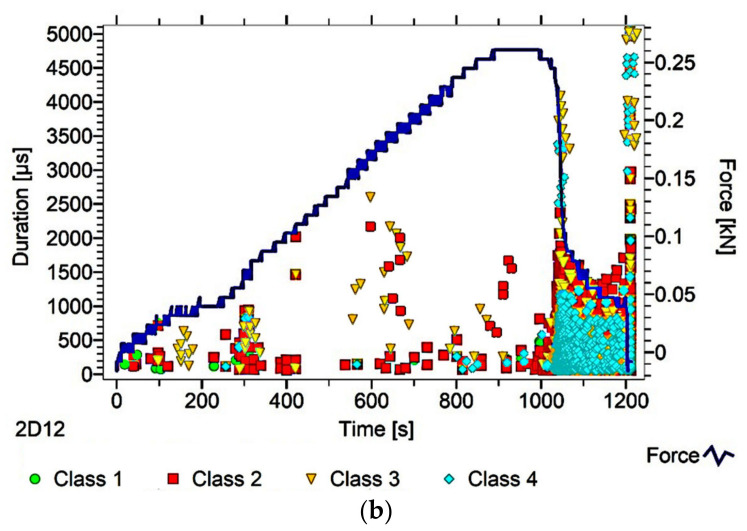
AE signal graphs for the mock BW sample: (**a**) signal strength distribution in time; (**b**) signal duration distribution in time, with plotted force increment curve.

**Figure 5:**
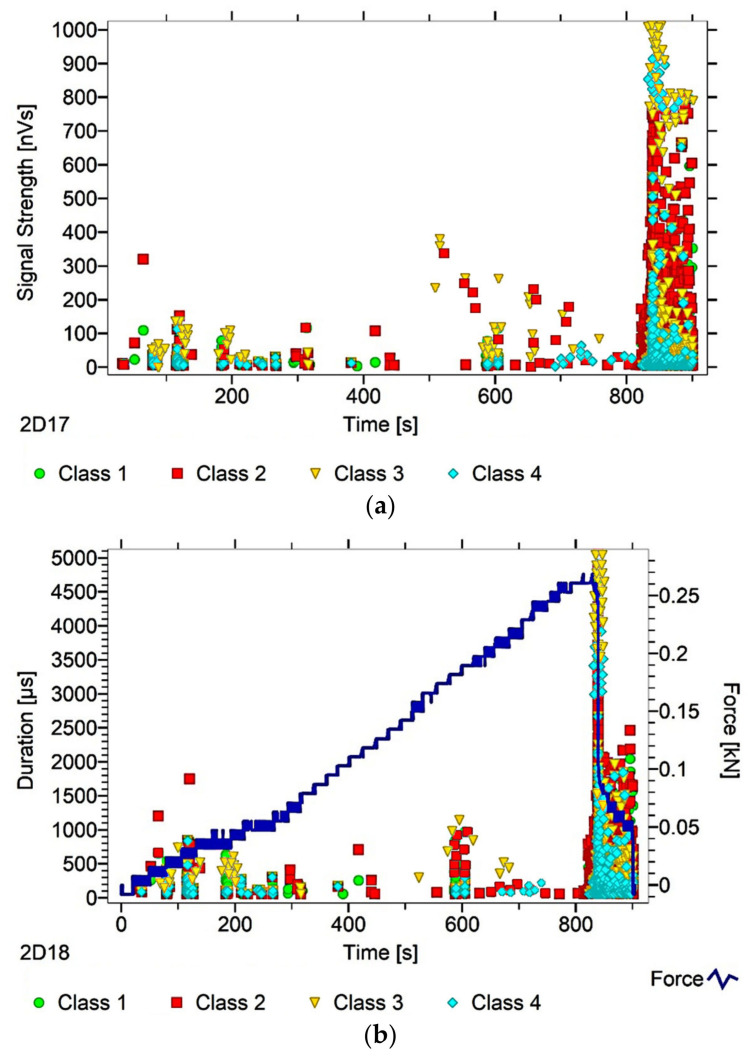
AE signal graphs for the mock BM sample: (**a**) signal strength distribution in time; (**b**) signal duration distribution in time, with plotted force increment curve.

**Figure 6:**
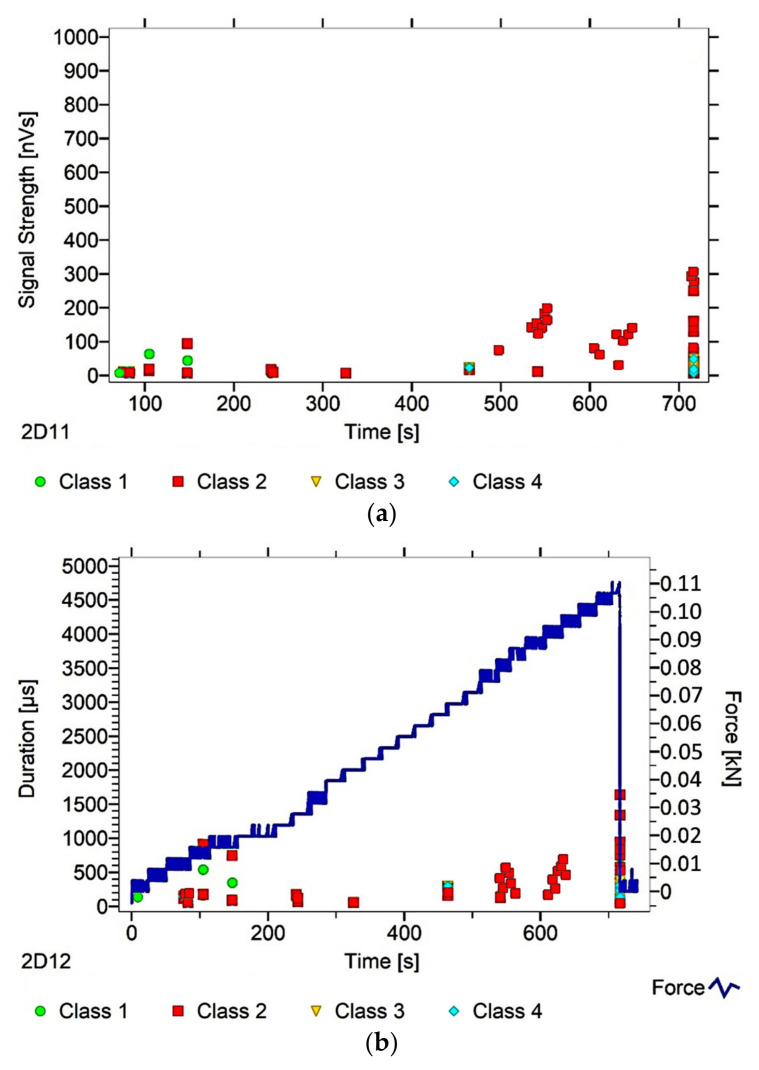
AE signal graphs for the mock BP sample: (**a**) signal strength distribution in time; (**b**) signal duration distribution in time, with plotted force increment curve.

**Figure 7:**
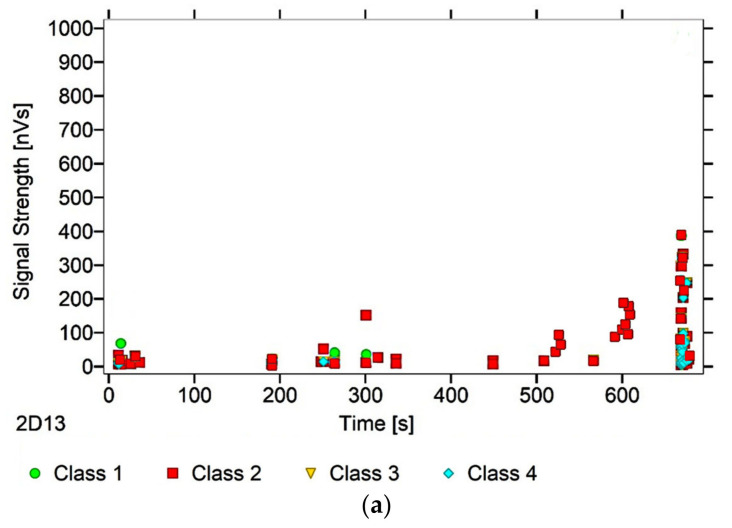

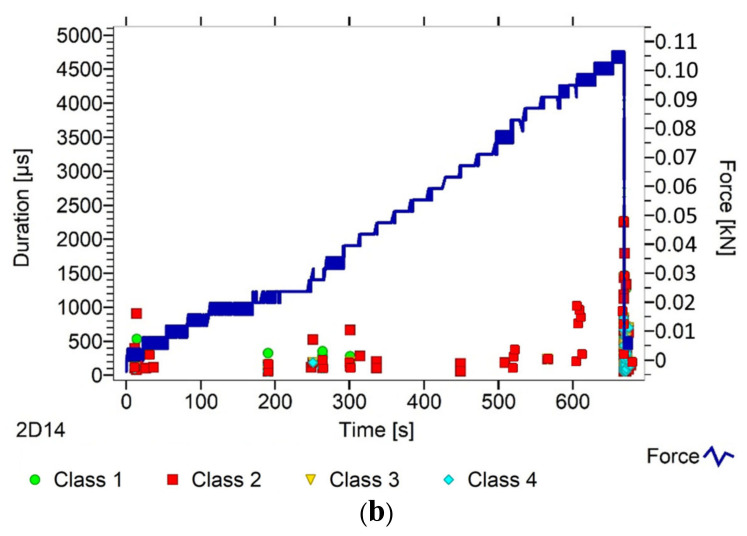
AE signal graphs for the mock BC sample: (**a**) signal strength distribution in time; (**b**) signal duration distribution in time, with plotted force increment curve.

Analysing Figure 3, Figure 4, Figure 5, Figure 6 and Figure 7, it can be seen that for samples in the air-dry state exposed to environmental factors (BS, BW and BM), the signals of 1–3 classes can be seen from the beginning of the external load action. Shortly after applying the force, Class 3 signals begin to appear, indicating that the fiber destruction process has begun. As the cracks in the tensile zone progress and the cracks deepens, Class 4 signals of gradual fiber breakage start to appear at the bottom of the sample. Exceeding the stress limits in the material results in a rapid increase in the recorded descriptors.

In the case of samples subjected to direct fire (BP) and high temperature (BC), two classes of signals were observed in the recorded time waveforms—Class 1 and Class 2. The authors related this fact to the possibility of the damage or degradation of the reinforcing fibers under the influence of temperature, which was associated with a change in the way the components are destroyed. It was found that dry samples and samples exposed to environmental factors, due to the presence of reinforcing fibers, were destroyed by exceedingly high bending stresses. On the other hand, the samples exposed to temperature cracked due to too much shear stress, likely because of the degradation of the fibers. To confirm these assumptions, a microscopic analysis of the fractures extracted from the tested components was conducted. During the analysis of the characteristics of the recorded descriptors, it was also found that during the bending of flamed and fired samples, the occurrence of most AE events was associated with a lower strength of signals than those found in the cases of samples from other groups. According to the authors, this fact confirms previous assumptions, which in their opinion validates the use of the AE method for assessing the degree of change of the mechanical parameters of the fiber–cement boards.

Analysing the classes of AE signals occurring in the analysed patterns for samples BP and BC, it was found that the absence of Class 3 and 4 signals in the recorded events clearly indicates damage to the reinforcing fibers, or the possibility of delamination and voids in the material structure. It should also be noted that there is a close correlation between the type of AE signal classes recorded, and the destructive force value. This gives rise to the conclusion that the use of the AE method may allow the analysis of fiber–cement components in terms of the occurrence of material defects already formed at the production stage (e.g., discontinuities, uneven distribution of reinforcing fibers).

In summary, it was found that in the case of samples in the air-dry state, soaked in water and cyclically frozen and defrosted, the process of sample destruction was ductile. The result of the bending force was a deepening and widening of the crack in the element’s tension zone. After reaching maximum strength, the samples were gradually unloaded. On the other hand, samples exposed to flames and high temperatures broke in a fragile manner. Along with reaching the maximum value of force, the element rapidly split into two parts. The differences in destruction mechanisms clearly influenced the types of recorded signal classes.

#### 3.1.2. Analysis of Frequencies Accompanying Changes in Mechanical Parameters

**Figure 8:**
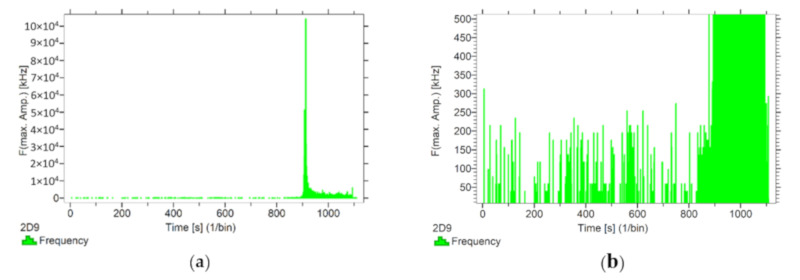
Frequency distribution graph during the test of the mock BS sample: (**a**) taking into account the entire frequency range; (**b**) specifying the frequency range before destruction.

**Figure 9:**
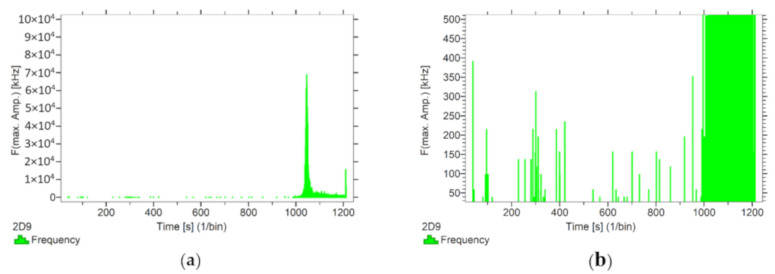
Frequency distribution graph during the test of the mock BW sample: (**a**) taking into account the entire frequency range; (**b**) specifying the frequency range before destruction.

**Figure 10:**
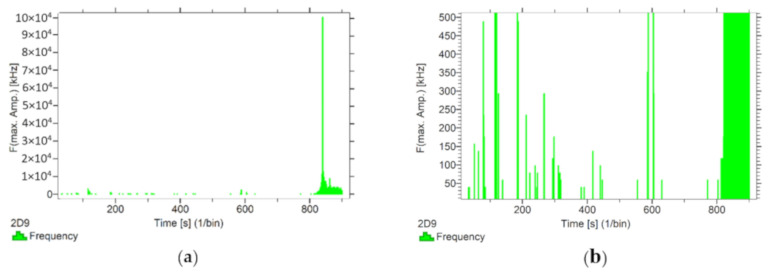
Frequency distribution graph during the test for the mock BM sample: (**a**) taking into account the entire frequency range; (**b**) specifying the frequency range before destruction.

**Figure 11:**
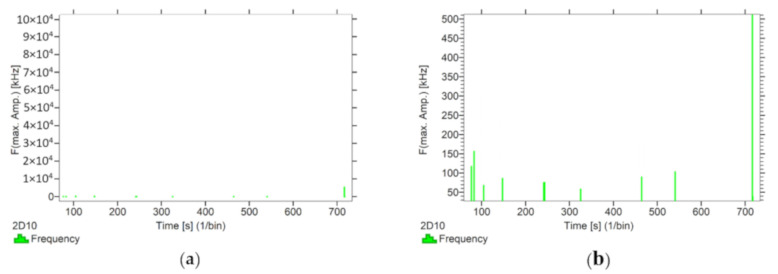
Frequency distribution graph during the test of the mock BP sample: (**a**) taking into account the entire frequency range; (**b**) specifying the frequency range before destruction.

**Figure 12:**
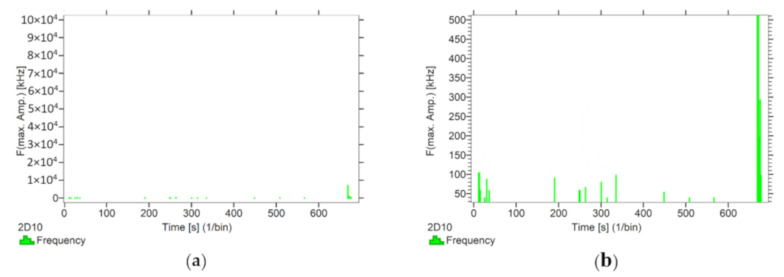
Frequency distribution graph during the test of the mock BC sample: (**a**) taking into account the entire frequency range; (**b**) specifying the frequency range before destruction.

Using the options available in the Vallen software, the frequencies accompanying the event emissions were extracted for the recorded data. The analysis results are presented in Figure 8, Figure 9, Figure 10, Figure 11 and Figure 12. For a more readable representation of the frequency ranges emitted by the material, two frequency distribution graphs are provided for each of the mock sample series. The first one is used to present the frequencies from the whole tested run, and the second one details the frequencies of signals recorded before the destruction moment.

Analysing the graphs presented above, it can be concluded that the results from subjecting the tested components to two groups of operating factors (environmental and exceptional) illustrated significant differences in the emitted frequency ranges. Changes in the mechanical parameters of the samples in the air-dry state, water-soaked and cyclically frozen then defrosted under an external load, are associated with low and high frequency signals. Most of the recorded frequencies exceed the 200 kHz threshold, and some events produce sounds of 300–500 kHz. The situation is different for flamed and fired samples. The bending of components exposed to high temperatures caused events at much lower frequencies, only some of which exceed 100 kHz.

### 3.2. Results of Microscopic Analyses

**Figure 13:**
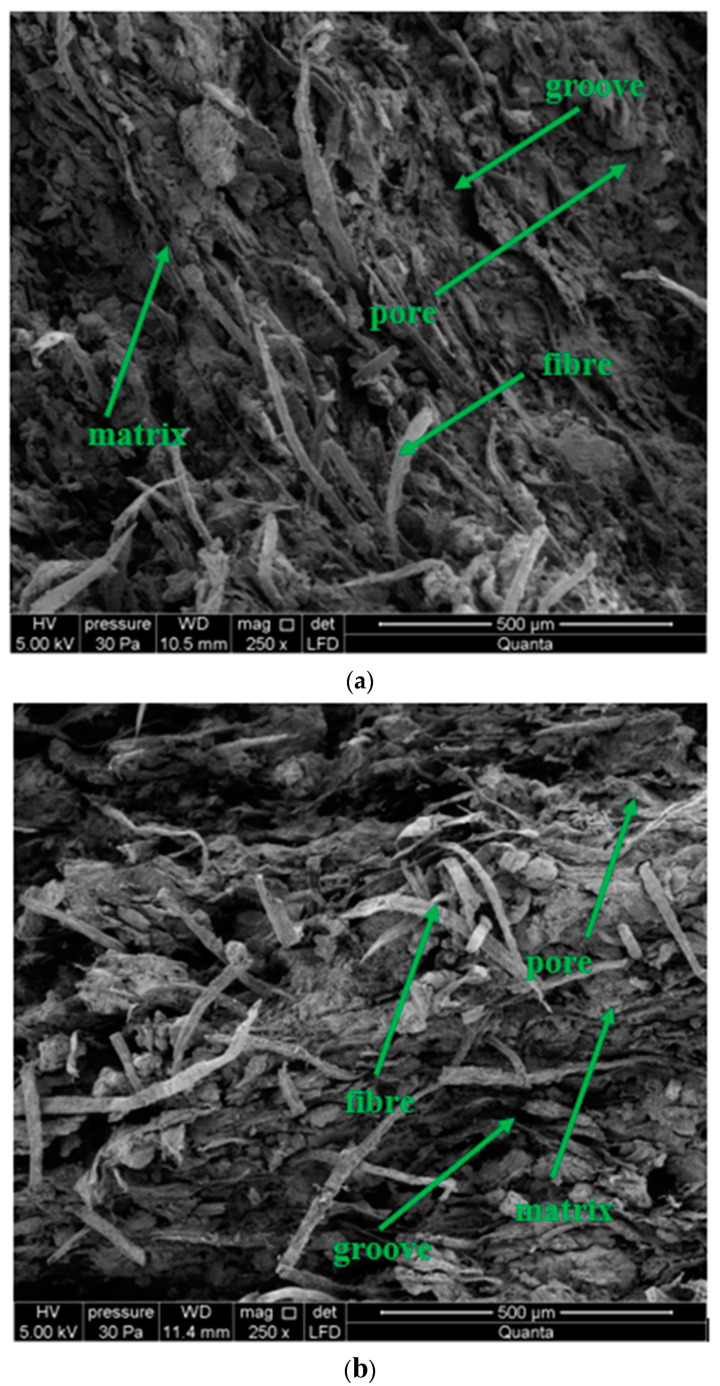

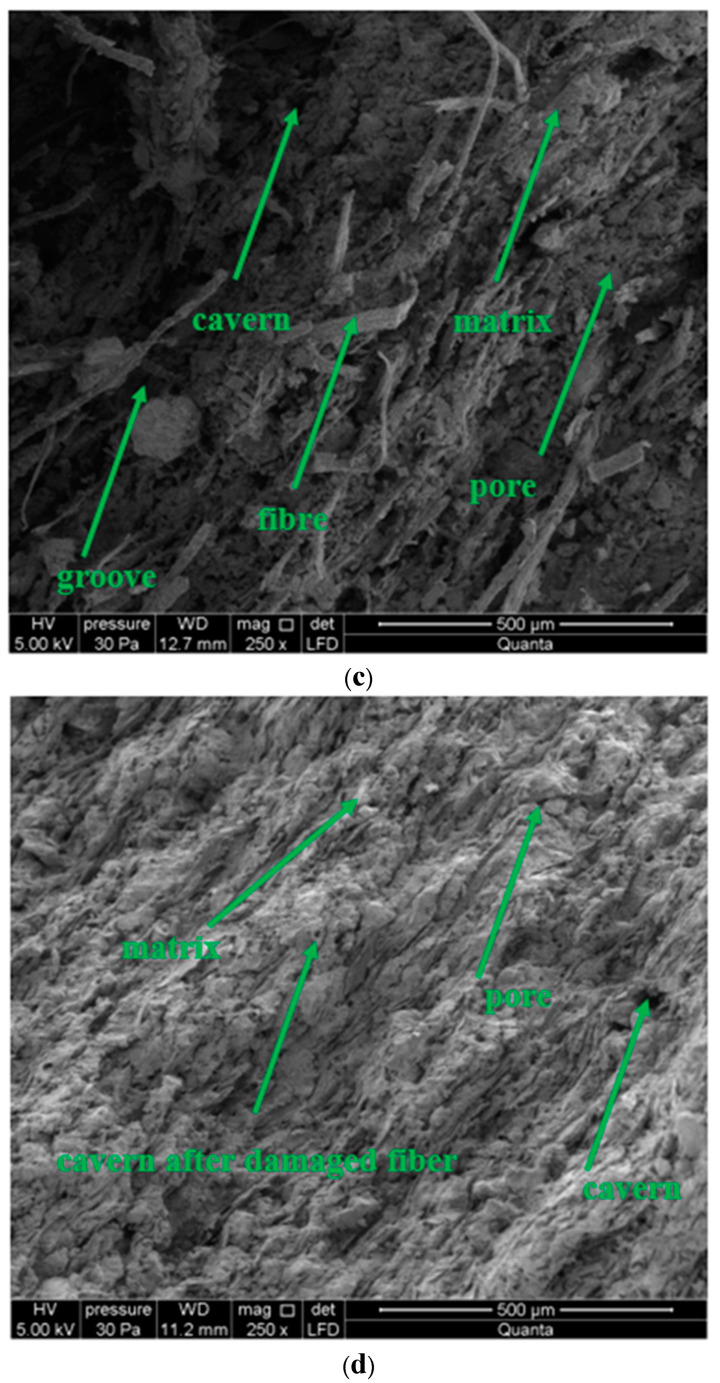

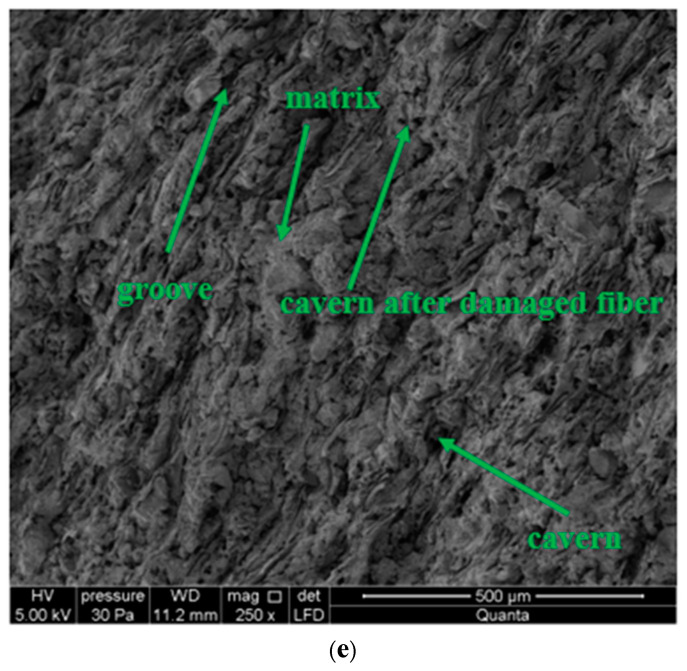
Image of the mock microstructure: (**a**) of BS sample, (**b**) of BW sample, (**c**) of BM sample, (**d**) of BP sample, and (**e**) of BC sample.

Microstructure images of the extracted sample fractures are shown in Figure 13. All images show a 250-fold magnification of the surface.

Based on the analysis of the results shown in Figure 13, it should be concluded that the macrostructure of each of the analysed board samples before microscopic examination was determined, visually, as compact. Microscopic observations determined that the structure of the samples were fine-porous, with pore size up to 50 µm. Deep grooves of up to 500 µm in width were found on the fracture surfaces. A high density of irregularly distributed cellulose fibers was observed at the tested fractures (Figure 13a–c), except for the flamed sample and the fired sample. In the burned sample, it was observed that most of the fibers are fired or blended into the matrix. Flaming causes gradual burning of fibers, and the degradation of their structure, depending on the flame range (Figure 13d,e). Caverns and grooves from the torn fibers were observed. The matrix structure is defined as granular with numerous delaminations. No space was observed between the fibers and the matrix, indicating a strong bond between them. In the analysis of the flamed sample (Figure 13d) a locally altered matrix structure was observed.

According to the authors, the microscopic observations performed confirmed previous assumptions, which justify the necessity and legitimacy of the AE method for evaluating the degree of change in the mechanical parameters of fiber–cement composites.

## 4. Discussion

Table 4, Table 5, Table 6, Table 7, Table 8 and Table 9 present a summary of the results of the tests. Analysing the data contained in them, it can be seen that the type of factor to which fiber–cement boards are exposed has a significant impact on the processes taking place inside its structure. According to the authors, this fact can be the basis for creating a professional system for assessing the condition of used boards.

An important element in the aforementioned system of intelligent supervision of the safety of the use of structures using fiber–cement elements will be the AE method. There is no information in the literature on the construction of such a system. Of course, there are scientific papers devoted to the use of the AE method as a research tool, but only to determine the phenomena occurring in a cement–fiber composite. In previous publications [57,58], the AE method has been used to assess the degree of destruction of boards that were previously affected by high temperatures. However, the analysis of registered descriptors without grouping the signals did not allow the direct connection of the AE signals with the endurance parameters of the plates, or the linking of them to specific destructive processes occurring in the structure. Therefore, these publications carried out additional analyses of registered AE events using artificial neural networks, which allowed for the initial division of events into signals corresponding to matrix cracking and fiber cracking. However, the results presented in this article show the possibility of creating an engineering system based on the pattern recognition method, i.e., creating a black box that can be operated by any engineer, not just a well-educated person. The use of the AE method allows one to monitor in real time, and intervene when the need arises, and not when a failure occurs.

## 5. Conclusions

This article presents a proposal for using the AE method to assess the condition of fiber–cement boards. The research was carried out on five groups of elements exposed to environmental and exceptional factors. During the three-point bending tests, AE signals were recorded, which were then grouped into four classes using the k-means algorithm. Based on the presented results, it was found that the indicated method can be successfully used to confirm or deny the presence, and to assess the condition of, reinforcing fibers in boards, which directly affect the strength parameters of these elements. The frequencies recorded before destruction were also traced for each sample. It has been noted that the frequency values are significantly different for samples with reinforcements, compared to elements with fired or degraded fibers. Initial hypotheses were confirmed during microscopic studies using a scanning electron microscope.

The following specific conclusions were drawn on the basis of the tests performed:

1. The implementation of the k-means grouping method based on the analysis of the AE signal parameters gives positive results in the classification of destructive processes taking place in the structure of fiber–cement boards.
2. The application of the k-means grouping method allows the distinguishing of the processes taking place in the structure of fiber–cement boards subjected to an external load.
3. Tracking events assigned to specific signal classes allows for evaluating the changes in the mechanical parameters of the material.
4. The presence of reinforcing fibers significantly affects the distribution and the number of AE signals of individual classes, and the strength of the boards.
5. The frequencies emitted by changes in the fiber–cement structure are closely linked to the presence of fiber reinforcement.
6. It was found that the application of the AE method enables the effective detection and monitoring of the initiation of changes in the structure, as well as the separation and identification of AE signals corresponding to different types of processes affecting the change of the mechanical parameters of fiber–cement boards.
7. The developed reference signal base provides a theoretical basis for the application of the AE technology, based on the standard for the detection and monitoring of cracks and delamination propagation in full-size fiber–cement components.

## Figures and Tables

**Table 1:** The conditioning and testing plan.

Sample Determination	Type of Conditioning	Sample Length [mm]	Sample Width [mm]	Sample Thickness [mm]	Number of Samples in the Group
BS	Air-dry state	300	50	8	5
BW	Soaking in water	300	50	8	5
BM	50 freezing–defrosting cycles	300	50	8	5
BP	A direct flame of a gas burner for 10 min	300	50	8	5
BC	Firing in a laboratory oven at 230 °C for 3 h	300	50	8	5

**Table 2:** Composition data of the tested board.

Manufacturer Information about Composition of Boards	Result of Observation and EDS Analysis
Portland cement	Presence confirmed
Mineral binders	Silicate binder
Natural organic reinforcing fibers	Cellulose fibers
Synthetic organic reinforcing fibers	PVA (Polyvinyl Alcohol) fibers

**Table 3:** Technical data of the tested board.

Parameter	Conditions	Value	Unit
Density	Dry condition	>1.65	g/cm^3^
Flexural strength	Perpendicular Parallel	>23 >18.5	N/mm^2^ N/mm^2^
Modulus of elasticity		12,000	N/mm^2^
Stretching at humidity	30–95%	1.0	mm/m
Porosity	0–100%	>18	%

**Table 4:** Summary of research results for BS samples.

Sample	Number of Class 1 Signals	Number of Class 2 Signals	Number of Class 3 Signals	Number of Class 4 Signals	Max. Signal Energy [eu]	Max. Force [kN]	MOR [MPa]	Max. Frequency before Destruction [kHz]
BS_1_	1747	8644	2040	2648	5049	0.29	27.19	316
BS_2_	2042	7256	2165	2543	5126	0.30	28.13	403
BS_3_	1635	7269	1847	2714	5098	0.29	27.19	359
BS_4_	1798	9842	2264	2796	4863	0.30	28.13	426
BS_5_	2249	11365	2073	2379	5194	0.30	28.13	511
Arithmetic average	1894.2	8875.2	2077.8	2616	5066	0.296	27.75	403
Standard deviation	221.74	1574.10	139.28	144.59	111.81	0.005	0.46	65.88

**Table 5:** Summary of research results for BW samples.

Sample	Number of Class 1 Signals	Number of Class 2 Signals	Number of Class 3 Signals	Number of Class 4 Signals	Max. Signal Energy [eu]	Max. Force [kN]	MOR [MPa]	Max. Frequency before Destruction [kHz]
BW_1_	2365	7424	2084	2215	5055	0.26	24.38	398
BW_2_	1823	7982	2152	2196	5139	0.28	26.25	452
BW_3_	1964	8214	1836	2364	5024	0.29	27.19	374
BW_4_	2159	6920	2174	1974	5097	0.28	26.25	512
BW_5_	2144	7563	2296	2259	4936	0.28	26.25	367
Arithmetic average	2091	7620.6	2108.4	2201.6	5050.2	0.278	26.06	420.6
Standard deviation	184.64	450.68	152.44	127.81	69.03	0.01	0.92	54.58

**Table 6:** Summary of research results for BM samples.

Sample	Number of Class 1 Signals	Number of Class 2 Signals	Number of Class 3 Signals	Number of Class 4 Signals	Max. Signal Energy [eu]	Max. Force [kN]	MOR [MPa]	Max. Frequency before Destruction [kHz]
BM_1_	1736	6422	1759	2168	5023	0.26	24.38	1023
BM_2_	2054	6948	2052	2232	5102	0.27	25.31	415
BM_3_	2148	6536	1866	2085	5074	0.26	24.38	504
BM_4_	1947	7049	2173	2266	5130	0.28	26.25	362
BM_5_	2072	7197	1945	1987	5069	0.26	24.38	388
Arithmetic average	1991.4	6830.4	1959	2147.6	5079.6	0.266	24.94	538.4
Standard deviation	142.93	299.82	143.73	101.26	35.75	0.008	0.75	246.98

**Table 7:** Summary of research results for BP samples.

Sample	Number of Class 1 Signals	Number of Class 2 Signals	Number of Class 3 Signals	Number of Class 4 Signals	Max. Signal Energy [eu]	Max. Force [kN]	MOR [MPa]	Max. Frequency before Destruction [kHz]
BP_1_	17	67	4	4	1823	0.11	10.31	113
BP_2_	25	84	3	8	1526	0.09	8.44	109
BP_3_	12	66	4	10	1465	0.10	9.38	126
BP_4_	10	72	7	9	1506	0.11	10.31	94
BP_5_	20	79	3	12	1764	0.9	8.44	116
Arithmetic average	16.8	73.6	4.2	8.6	1616.8	0.10	9.38	111.6
Standard deviation	5.42	6.95	1.47	2.65	146.80	0.009	0.84	10.44

**Table 8:** Summary of research results for BC samples.

Sample	Number of Class 1 Signals	Number of Class 2 Signals	Number of Class 3 Signals	Number of Class 4 Signals	Max. Signal Energy [eu]	Max. Force [kN]	MOR [MPa]	Max. Frequency before Destruction [kHz]
BC_1_	20	70	4	4	1885	0.15	14.06	113
BC_2_	14	84	2	10	1624	0.10	9.38	135
BC_3_	10	112	1	11	1894	0.09	8.44	92
BC_4_	23	62	4	6	1733	0.11	10.31	84
BC_5_	11	97	2	8	2014	0.11	10.31	141
Arithmetic average	15.6	85	1.44	6.56	1830	0.11	10.5	113
Standard deviation	5.08	18.04	1.2	2.56	136.25	0.02	1.91	22.58

**Table 9:** Summary of research results.

Sample	Average Number of Class 1 Signals	Average Number of Class 2 Signals	Average Number of Class 3 Signals	Average Number of Class 4 Signals	Average Max. Signal Energy [eu]	Average Max. Force [kN]	Average MOR [MPa]	Average Max. Frequency before Destruction [kHz]
BS	1894.2	8875.2	2077.8	2616	5066	0.296	27.75	403
BW	2091	7620.6	2108.4	2201.6	5050.2	0.278	26.06	420.6
BM	1991.4	6830.4	1959	2147.6	5079.6	0.266	24.94	538.4
BP	16.8	73.6	4.2	8.6	1616.8	0.10	9.38	111.6
BC	15.6	85	1.44	6.56	1830	0.11	10.5	113
